# Clinical application of artificial intelligence-assisted three-dimensional planning in direct anterior approach hip arthroplasty

**DOI:** 10.1007/s00264-023-06029-9

**Published:** 2023-11-15

**Authors:** Weihua Yang, Tianyi Gao, Xingyu Liu, Kaiwei Shen, Feitai Lin, Yan Weng, Bei Lin, Deng Liang, Eryou Feng, Yiling Zhang

**Affiliations:** 1 Department of Arthrosis Surgery, Fuzhou Second Hospital, Fuzhou, China; 2 Longwood Valley MedTech, No.2 Ronghua South Road, Daxing District, Beijing, China; 3 Department of Arthrosis Surgery, Fujian Medical University Union Hospital, No. 29, Xinquan Road, Gulou District, Fuzhou, China

**Keywords:** Total hip arthroplasty, 3D preoperative planning, Artificial intelligence

## Abstract

**Purpose:**

The objective of this study was to investigate the efficacy of an artificial intelligence-assisted 3D planning system (AIHIP) in total hip arthroplasty by direct anterior approach and assess the reliability of the AIHIP preoperative program in terms of both interobserver and intraobserver agreement.

**Methods:**

A retrospective analysis was conducted on patients who underwent unilateral primary THA via direct anterior approach from June 2019 to March 2022. Participants were randomly assigned to receive either the AIHIP system (*n* = 220) or the 2D template (control group) (*n* = 220) for preoperative planning. The primary outcome aimed to evaluate the correspondence between the prosthesis selected intro-operation and the one planned preoperatively, as well as to calculate the intraclass correlation coefficient (ICC). Secondary outcomes included operation time, intraoperative blood loss, fluoroscopy times, Harris hip score (HHS), lower limb length difference (LLD), femoral offset (FO), and bilateral femoral offset difference.

**Results:**

No significant differences were observed in gender, age, body mass index (BMI), aetiology, and American Society of Anesthesiologists (ASA) score between the two groups. Both planning methods exhibited good intraobserver agreement for component planning (ICC: 0.941–0.976). Interobserver agreement for component planning was comparable between the two methods (ICC: 0.882–0.929). In the AIHIP group, the accuracy of acetabular cup and femoral stem prosthetics planning significantly improved, with accuracies within the size range of ± 0 and ± 1 being 76.8% and 90.5% and 79.5% and 95.5%, respectively. All differences between two groups were statistically significant (*p* < 0.05). Patients receiving AIHIP preoperative planning experienced shorter operation times, reduced intraoperative blood loss, fewer fluoroscopy times, and lower leg length discrepancy (LLD) (*p* < 0.05). Moreover, they demonstrated a higher Harris hip score (HHS) at three days post-surgery (*p* < 0.05). However, no significant differences were found in femoral offset (FO), difference of bilateral femoral offsets, and HHS at 1 month after the operation.

**Conclusion:**

Utilizing AIHIP for preoperative planning of direct anterior approach THA can significantly enhance the accuracy of prosthetic sizing with good reliability, decrease operation time, reduce intraoperative blood loss, and more effectively restore the length of both lower limbs. This approach has greater clinical application value.

## Introduction

Total hip arthroplasty (THA) serves a crucial function in reestablishing the normal anatomical relationships and reconstructing the normal biomechanics of the hip joint [1]. Preoperative planning enables the testing of prosthesis models and simulates their placement in advance, which is vital for enhancing the efficiency, accuracy, and safety of the operation. At present, domestic preoperative planning primarily relies on two-dimensional templates [2], but their accuracy is relatively suboptimal in clinical practice due to limitations in shooting angles, magnification errors [3], and inadequate shooting postures [4]. These issues render traditional two-dimensional (2D) template-based preoperative planning less accurate [5]. The mismatch of intraoperative prostheses may result in prolonged operation time, increased intraoperative trauma, and consequently impact the surgical outcome, potentially even leading to surgical failure necessitating revision.

In recent years, artificial intelligence (AI) technology has advanced rapidly. AIHIP software (version 3.0, Longwood Valley Technology, China), which incorporates AI technology, can automatically recognize CT images, swiftly construct three-dimensional models of the hip joint, intelligently match prosthetic sizes, and aid medical staff in preoperative planning to increase the success rate of surgery [6]. AIHIP has been shown to improve the precision of preoperative templating and efficiency in total hip replacement using the posterolateral approach [6, 7]. However, the accuracy and reliability of AIHIP in different total hip replacement approaches still require further validation. This study represents the first investigation of AIHIP’s clinical applicability in direct anterior approach (DAA) total hip replacement.

The aim of this study was to compare the AIHIP software with 2D templates for preoperative prediction of total hip replacement prosthesis size and to evaluate the accuracy and clinical efficacy of the artificial intelligence 3D programming system in preoperative DAA planning prosthesis. The hypothesis is that the AIHIP system will provide more precise prosthetic size planning and better guidance for anatomical recovery.

## Materials and methods

This study involved a retrospective analysis of patients who underwent THA treatment using the direct anterior approach at the Second Hospital of Fuzhou from June 2019 to March 2022. Inclusion criteria were as follows: ① Primary THA for unilateral hip disease; ② Pinnacle acetabular cup and Tri-Lock femur (DePuy Orthopaedics, USA) were utilized; ③ relevant clinical data were complete. Exclusion criteria included the following: ① patients who did not undergo surgery after preoperative planning; ② preoperative images that did not conform to preoperative planning criteria; ③ presence of active infected lesions in the hip joint or other parts of the body prior to surgery; ④ discontinuation or absence of follow-up data. Based on these criteria, 440 patients (193 men and 247 women) with a mean age of 57.16 years (range: 47 to 76 years) and a mean BMI of 23.61 kg/m^2^ (range: 15 to 31 kg/m^2^) were enrolled. The patient population included 177 cases of femoral head necrosis, 103 cases of femoral neck fracture, 80 cases of hip osteoarthritis, and 80 cases of developmental dysplasia of the hip.

The study was approved by the Medical Ethics Committee of Fuzhou No. 2 Hospital (No. 20220109), and informed consent was obtained from all participating patients.

Limb length discrepancy (LLD) was defined as the vertical distance from the tip of the lesser trochanter on both sides to the line of the inferior border of the teardrop on both sides. Femoral offset (FO) was defined as the vertical distance from the centre of rotation of the hip joint to the longitudinal axis of the femoral stem.

### AIHIP preoperative planning

Collect hip CT image from 440 patients, including femoral head necrosis (ON), femoral neck fracture, dysplasia of the hip (DDH), hip osteoarthritis (OA), and other diseases of the hip. The CT scan ranged from the entire pelvis to 12 cm below the lesser trochanter of the femur with section thickness of 0.8 mm. Scan data is imported into AIHIP software in DICOM format.

#### Intelligent planning acetabular side

**Fig. 1:**
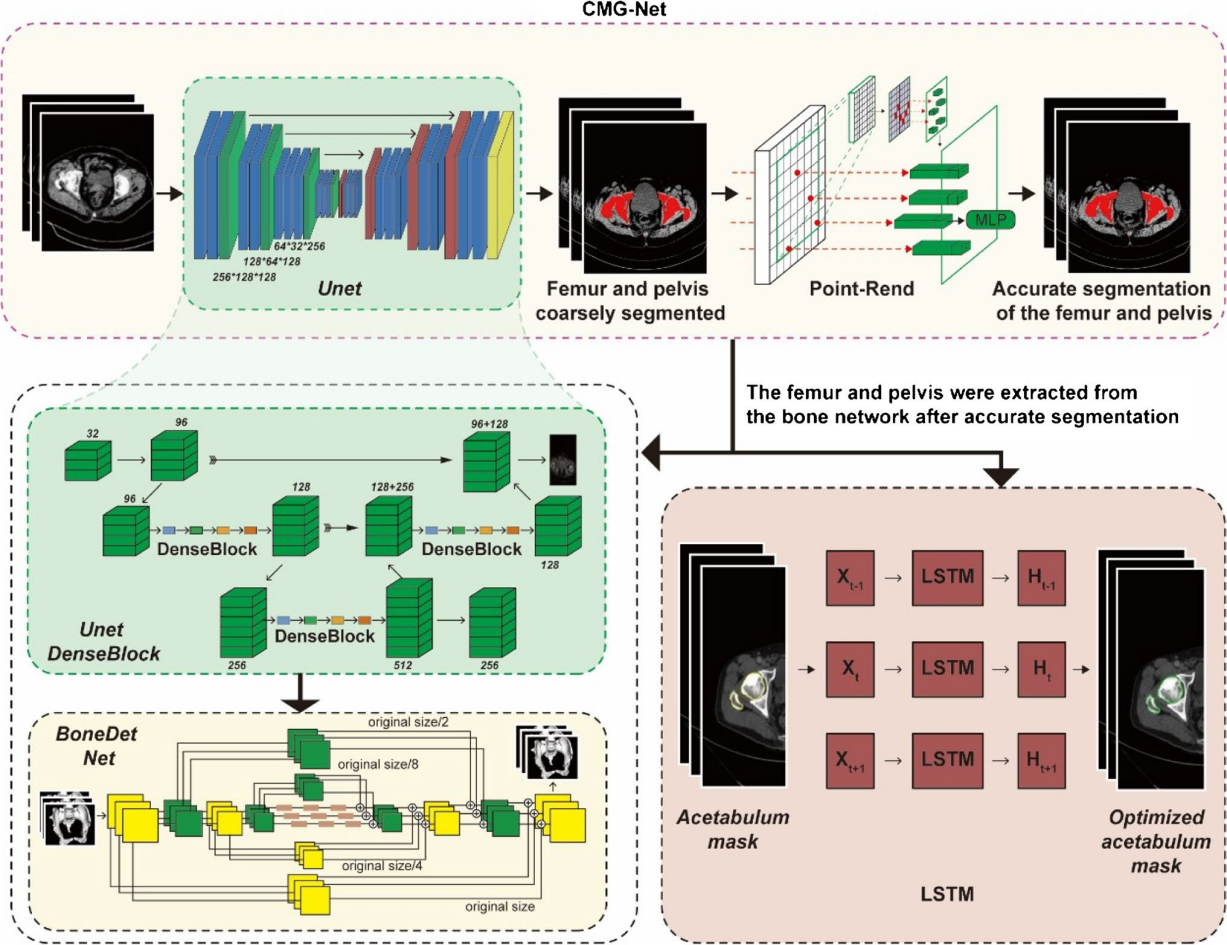
CMG-NET neural network technology

**Fig. 2:**
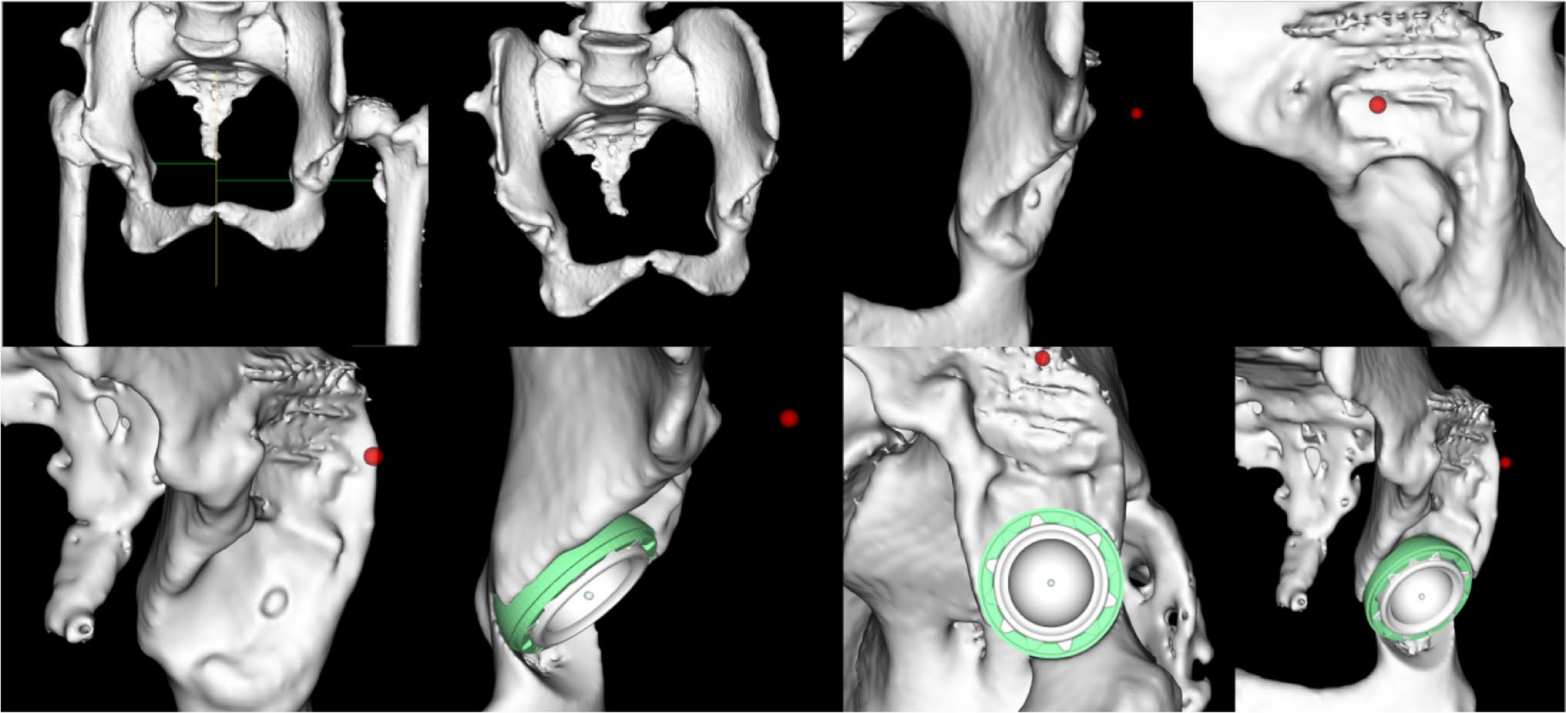
3D reconstruction of the pelvis position of the acetabular prosthesis is intelligently placed to calculate the most suitable angle

AIHIP system adopts the original CMG-NET neural network technology (Fig. 1), contributing to accurate clarification and segment of the acetabular anatomical structure by integrating Unet structure to perform first image recognition and segmentation of pelvis and femur, DenseBlock structure to smoothing fusion edge by the second segmentation, LSTM network to confirm the classification of acetabular side and femur side, and BoneDet network and PointRend technology to final confirm the defects on the acetabular side and femur side were identified by secondary image recognition. Then, based on key anatomical points, the acetabular structure is accurately reconstructed, the pelvis and both lower limbs are automatically corrected, and the anteroposterior diameter of the acetabulum is measured on the transverse position and the pelvis model, and the position of the acetabular prosthesis is intelligently placed to achieve the most suitable position and size of the acetabular component. Inclination, anteversion, and coverage of the acetabular component were planned as well (Fig. 2).

#### Intelligent planning of the femoral side

**Fig. 3:**
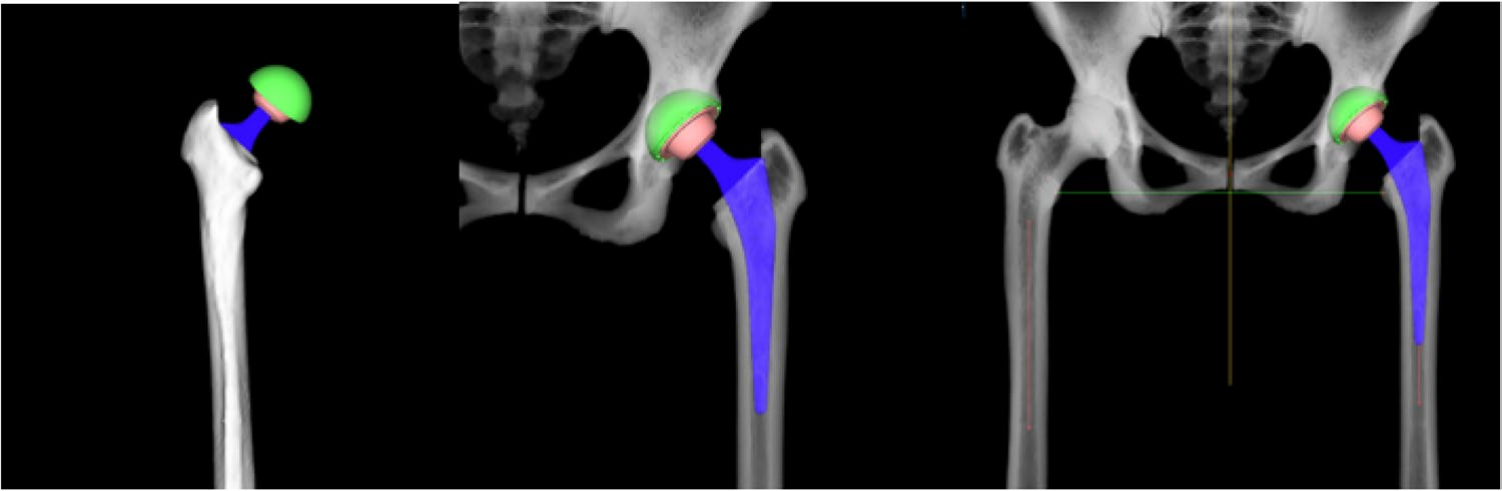
Femoral medullary cavity, anteversion angle of the femur, and the position of the femoral stem are automatically planned

**Fig. 4:**
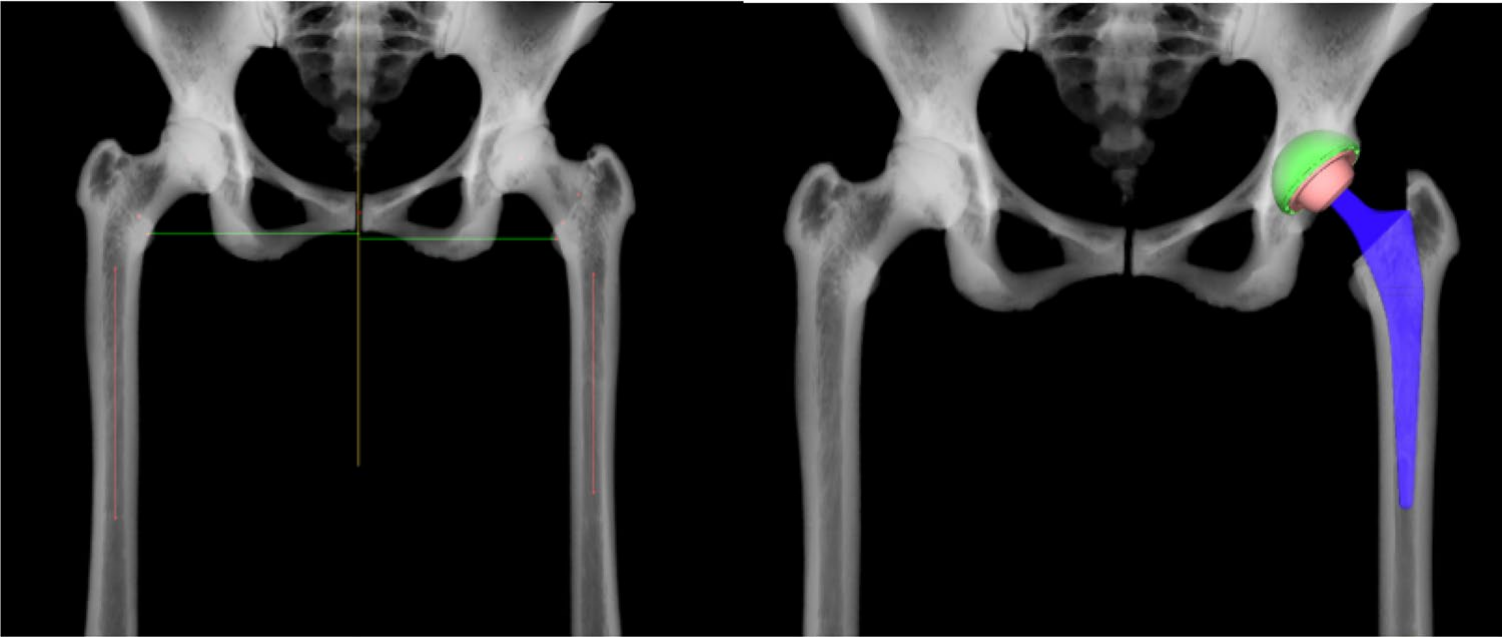
Postoperative X-ray simulation compared to preoperative X-ray

According to the calculated diameter of the femoral medullary cavity and the anteversion angle of the femur, the position of the femoral stem is planned by adopt segmentation module and prosthesis parameters of AHIP (Fig. 3), and the size of femoral head is matched on the basis of the difference between the lengths of lower limbs and the femoral offset as well as the level of femoral resection was determined by calculating the difference between contacting area according to coated line of the stem using Boolean operation. Moreover, AIPHIP software can enumerate the difference in the length of the lower limbs and the difference in the offset of the bilateral femurs after the placement of the femoral stem prosthesis according to the previous changes of the anatomical landmarks; also, it has good postoperative X-ray simulation ability to compare with preoperative X-ray (Fig. 4).

### Preoperative planning

To assess intra- and interobserver reliability, digital template planning and software planning were performed by two independent investigators before surgery. The same data were template planned again by both investigators two weeks later to minimize recall bias. Both groups were conducted by two experienced joint surgeons who were not involved in the preoperative planning process, ensuring that the preoperative planning of the prosthesis did not influence the intraoperative prosthesis selection.

Observation group: Patients received preoperative pelvic CT plain scans, with the scanning range extending from the highest point of the iliac spine to 12 cm below the lesser trochanter of the femur, and a scanning layer thickness of 0.8 mm. The scanned data were imported into AIHIP software in DICOM format, and the software automatically constructed acetabular and femur structures, measured anatomical data, and matched appropriate prostheses.

Control group: Patients underwent preoperative imaging using the same radiographic machine with standard anterior–posterior pelvic radiographs. The patient was placed in a supine position, both lower extremities internally rotated, with the big toes touching each other and the patella facing forward. A uniform magnification of 1.15 was determined, and the prosthesis type was measured by the same operator using a film template.

### Surgical methods

The patient was placed supine on a radiolucent operating table. The surgical incision began approximately 2–3 cm lateral and 2–3 cm distal to the anterior superior iliac spines and extended approximately 6 ~ 8 cm toward the fibular head. The surgeon separated the gap between the sartorius muscle and the tensor fascia lata muscle and entered the Heuter interval. The capsulotomy was performed to expose the femoral neck, and the femoral neck was resected according to the preoperative planning osteotomy height. After the femoral head was removed, the capsule around the acetabulum was released, and the acetabular labrum was resected. The acetabulum was concentrically reamed according to technique instruction. The appropriate type of acetabular cup and liner was inserted based on the plan and actual situation. Referring to the preoperative planning, capsule and co-joined tendon around proximal femur were entirely released in order to lift proximal femur. Then, femoral canal was broached, and the stem antevision was ascertained in order to implant the real femoral prosthesis. The hip stability was checked to require the maximum of range of motion. Finally, the capsule was sutured as well as possible.

### Result indicators

The following indicators were compared between the two groups: prosthesis matching rate; operation time; intraoperative blood loss; fluoroscopy times; Harris hip score (HHS); bilateral lower limb length difference (LLD); femoral offset (FO); and the difference in femoral offset between the two sides.

### Statistical methods

SPSS 23.0 statistical software was used for data analysis. Measurement data were expressed as mean ± standard deviation, and an independent sample *t*-test was used for comparison between groups. The χ^2^ test and rank sum test were used for comparing count data between groups. Due to non-normal distribution, fluoroscopy times were compared using the Mann–Whitney *U* test and expressed as median and interquartile range (IQR). Intraclass correlation coefficients (ICCs) were used to assess the reliability of intraobserver and interobserver repeated measures. ICC values less than 0.5 represented weak agreement, between 0.5 and 0.75 represented moderate agreement, between 0.75 and 0.9 represented strong agreement, and greater than 0.9 represented very strong agreement [8]. A *p*-value less than 0.05 was considered statistically significant.

## Results

### Patient characteristics

This study included a total of 440 patients. The observation group consisted of 220 patients with a mean age of 56.6 ± 14.5 years (range 47 to 76 years), comprising 92 males and 128 females, 91 cases of femoral head necrosis, 50 cases of femoral neck fracture, 44 cases of osteoarthritis, and 35 cases of hip dysplasia. The control group consisted of 220 patients with a mean age of 57.4 ± 15.2 years (range 50 to 75 years), including 101 males and 119 females, 86 cases of femoral head necrosis, 53 cases of femoral neck fracture, 36 cases of osteoarthritis, and 45 cases of hip dysplasia. No statistically significant differences were found between the two groups in terms of general characteristics or aetiology (Table 1).

**Table 1:** Comparison of general data between the two groups

	Observation group (*n* = 220)	Control group (*n* = 220)	*p*
Gender			0.569
Male	92(41.8%)	101(45.9%)	
Female	128(58.2%)	119(44.1%)	
Age (years)	56.6 ± 14.5	57.4 ± 15.2	0.765
BMI			0.594
Underweight (< 18.50 kg/m2)	27 (12.2%)	23 (10.5%)	
Normal (18.50–24.99 kg/m2)	94 (42.7%)	90(40.9%)	
Overweight (25.00–29.99 kg/m2)	86 (39.1%)	87(39.5%)	
Obesity (≥ 30.00 kg/m2)	13(6%)	20(9.1%)	
Etiology			0.517
Necrosis of femoral head	91 (41.4%)	86 (39.1%)	
Fracture of femoral neck	50 (22.7%)	53 (24.1%)	
OA	44 (20%)	36 (16.4%)	
DDH	35 (15.9%)	45 (20.4%)	
Hartofilakidis type A	28 (12.7%)	33 (15%)	
Hartofilakidis type B	5 (2.2%)	8 (3.6%)	
Hartofilakidis type C	2 (1%)	4 (1.8%)	
Side (patient numbers)			0.519
Right	114 (51.8%)	103 (46.8%)	
Left	106 (48.2%)	117 (53.2%)	
ASA score			0.362
1	156 (70.9%)	168 (76.4%)	
2	64 (29.1%)	52 (23.6%)	

*ASA*, American Society of Anesthesiologists Classification of Physical Conditions; *BMI*, body mass index; *OA*, osteoarthritis; *DDH*, hip dysplasia

### Comparison of surgical results

**Fig. 5:**
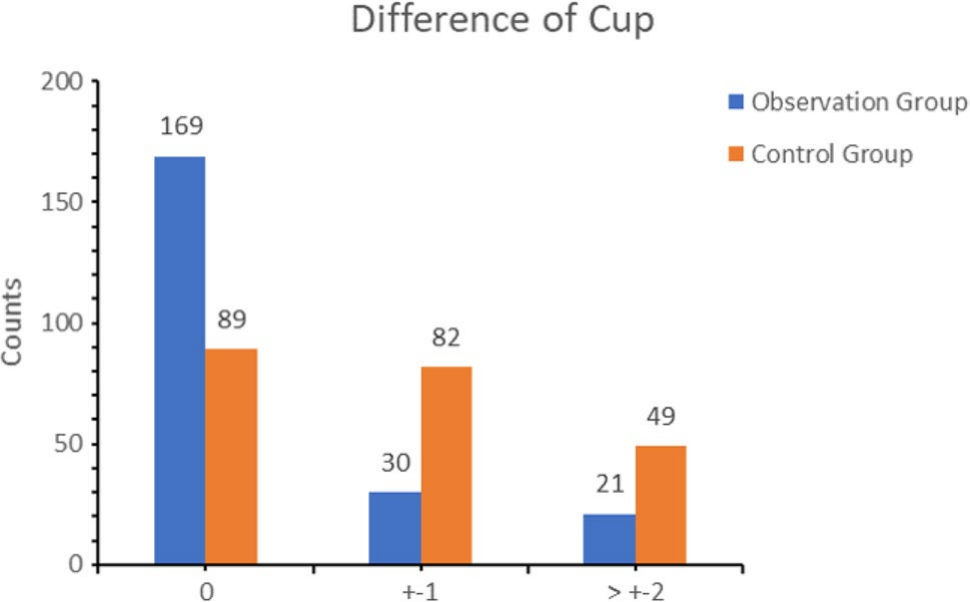
Templating accuracy of cup size

**Fig. 6:**
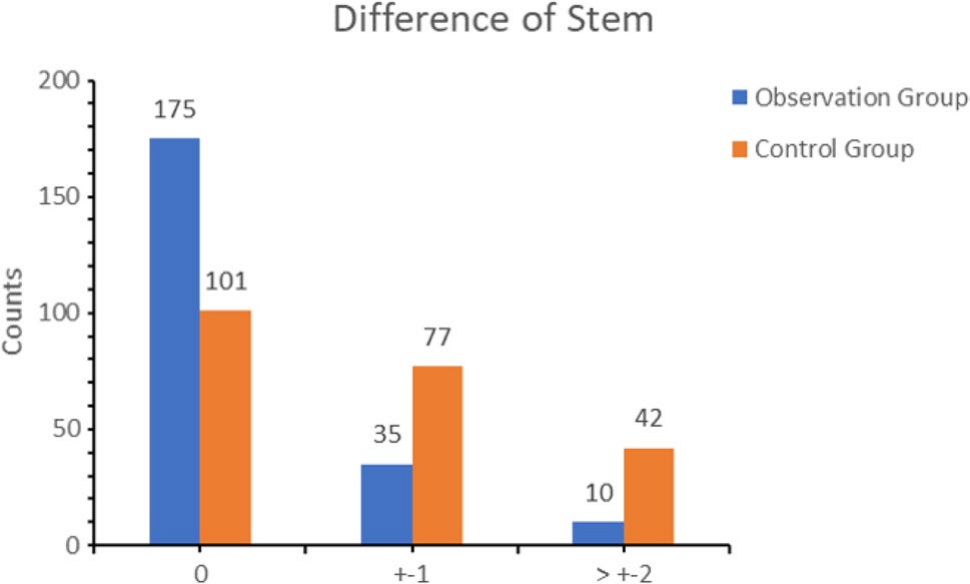
Templating accuracy of stem size

Prosthesis matching degree: In the observation group, 169 cases demonstrated complete matching between the postoperative acetabular cup size and preoperative planning, 30 cases showed general matching (± 1 size), and 21 cases exhibited mismatch (± 2 size and above). The conformity rates within the size range of ± 0 and ± 1 were 76.8% and 90.5%, respectively. For the femoral stem type matching, 175 cases were complete matches, 35 cases were general matches (± 1 size), and 10 cases were mismatches (± 2 size and above). The coincidence rate within the size range of ± 0 and ± 1 was 79.5% and 95.5%, respectively. In the control group, 89 cases exhibited complete matching between postoperative acetabular cup size and preoperative planning, 82 cases showed general matching (± 1 size), and 49 cases demonstrated mismatch (± 2 size and above). The conformity rates within the size range of ± 0 and ± 1 were 40.5% and 77.7%, respectively. For the femoral stem type matching, 101 cases were complete matches, 77 cases were general matches (± 1 size), and 42 cases were mismatches (± 2 size and above). The coincidence rate within the size range of ± 0 and ± 1 was 45.9% and 80.9%, respectively. The difference between the groups was statistically significant (*p* < 0.05) (Figs. 5 and 6, Table 2). The interobserver ICC for the acetabular template in the AIHIP group was 0.887 (95% CI, 0.786–0.942), and the intraobserver ICC was 0.962 (95% CI, 0.927–0.981). For the femoral template, the interobserver ICC was 0.929 (95% CI). In the 2D group, the interobserver ICC was 0.914 (95% CI, 0.836–0.956), and the intraobserver ICC was 0.948 (95% CI). The interobserver ICC for the femoral template was 0.882 (95% CI, 0.778–0.939), and the intraobserver ICC was 0.941 (95% CI, 0.885–0.970) (Table 3).

**Table 2:** Primary outcome measures

	Observation group (*n* = 220)	Control group (*n* = 220)	*p*
Acetabular side fit			< 0.001
Exact match	169 (76.8%)	89 (40.4%)	
General match	30 (13.6%)	82 (37.3%)	
Don’t match	21 (9.6%)	49 (22.3%)	
Femoral side fit			< 0.001
Exact match	175 (79.5%)	101 (45.9%)	
General match	35 (15.9%)	77 (35.0%)	
Don’t match	10 (4.6%)	42 (19.1%)

**Table 3:** Intra- and inter-observer ICC values for 2D and AIHIP planning methods

	ICC value	95% confidence interval	
		Lower	Upper
Interobserver agreement for 2D			
Acetabular	0.914	0.836	0.956
Femoral	0.882	0.778	0.939
Intraobserver agreement for 2D			
Acetabular	0.948	0.900	0.974
Femoral	0.941	0.885	0.970
Interobserver agreement for 3D			
Acetabular	0.887	0.786	0.942
Femoral	0.929	0.861	0.964
Intraobserver agreement for 3D			
Acetabular	0.962	0.927	0.981
Femoral	0.976	0.953	0.988

General surgical outcomes: The observation group experienced significantly lower intraoperative blood loss (229.81 ± 97.69 ml) compared to the control group (267.17 ± 113.88 ml) (*p* < 0.05). The number of intraoperative fluoroscopies was 0 (0,0) in the observation group and 1 (1,2) in the control group, indicating that the observation group had fewer intraoperative fluoroscopies than the control group (*p* < 0.001). The operative time for the observation group (43.22 ± 12.65 min) was shorter than that of the control group (68.36 ± 14.45 min) (Table 4).

**Table 4:** Secondary outcome measures

Factors	Observation group (*n* = 220)	Control group (*n* = 220)	*p*
Operation time (min)	43.22 ± 12.65	68.36 ± 14.45	< 0.001
Blood loss (ml)	229.81 ± 97.69	267.17 ± 113.88	0.031
Number of intraoperative fluoroscopy	0 (0.0)	1 (1.2)	< 0.001
LLD (mm)	0.71 ± 0.54	1.14 ± 0.59	< 0.001
FO (mm)	36.79 ± 7.94	37.92 ± 6.77	0.342
Offset difference after operation (mm)	3.73 ± 0.65	3.89 ± 0.68	0.160
HHS			
Preoperative	49.55 ± 3.55	49.08 ± 3.09	0.385
3 days after surgery	78.66 ± 3.32	74.01 ± 3.42	< 0.001
1 month after surgery	85.51 ± 3.69	84.88 ± 2.68	0.232

*FO* femoral offset, *LLD*, leg length discrepancy, *HHS* Harris hip score

Imaging and hip function scores: The LLD in the observation group was 0.71 ± 0.54 mm, while the LLD in the control group was 1.14 ± 0.59 mm. The accuracy of restoring leg length in both lower limbs was higher in the observation group (*p* < 0.001). The FO for the observation group was 36.79 ± 7.94 mm, and the FO for the control group was 37.92 ± 6.77 mm, with no statistically significant difference between the two groups. The postoperative offset difference was 3.73 ± 0.65 mm in the observation group and 3.89 ± 0.68 mm in the control group, and the difference was not statistically significant. The HHS score was higher in the observation group (78.66 ± 3.32) than in the control group (74.01 ± 3.42) at three days postoperatively (*p* < 0.001). However, at one month postoperatively, there was no difference between the HHS score in the observation group (85.51 ± 3.69) and the control group (84.88 ± 2.68) (Table 4).

## Discussion

This study compared the outcomes of AIHIP and 2D templates in patients undergoing unilateral DAA-THA, revealing several findings: (1) AIHIP was significantly more accurate in planning femoral and acetabular side prostheses compared to 2D templates. (2) Patients in the AIHIP group had shorter operative times, less intraoperative bleeding, and fewer intraoperative fluoroscopies, and AIHIP was more successful in reducing postoperative LLD compared to 2D templates. (3) Patients in the AIHIP group had higher HSS scores at three days postoperatively compared to 2D templates.

Previous studies have identified the difficulty of femoral exposure as a major challenge in the direct anterior approach to THA. The pursuit of adequate femoral exposure and excessive medullary reaming contributes to the occurrence of intraoperative fractures [9]. Some studies [10, 11] showed that most femoral complications were fractures of the calcar and the greater trochanter. Jewett et al. [12] described the prospect of using a fracture table to reduce the risk of femoral fracture in direct total approach hip replacement. The advantage of the DAA traction table, which can fix patients' lower limbs, reduces the incidence of complications of unequal lower limb lengths [13]. However, the current literature has raised much controversy about the time efficiency of intraoperative traction table [14, 15]. The difficulty in femoral exposure poses a threat to the undersized or malalignmental femoral stem implantation by the direct anterior approach (DAA), resulting in the risk of loosening and subsidence of the femoral stem [16]. The risk of femoral subsidence after THA has been reported to be 4–56.7% [17–20]. The AIHIP system can accurately segment and reconstruct the hip joint structure based on CT data through neural network technology and use intelligent algorithms to match the femoral stem prosthesis model, selecting the appropriate alignment and antevision. Chen et al. [21] reported that using AIHIP to predict the accuracy of the femoral stem is over 90%. It is believed that the AIHIP system can adjust the morphology of the femoral stalk in the medullary cavity and the degree of filling through various angles, which largely compensates for the shortcomings of the 2D template, which is limited in measuring the angle and cannot accurately measure the diameter of the medullary cavity, ultimately improving the prosthesis matching rate.

The direct anterior approach (DAA) accesses the hip socket through the muscle intervals, which preserves the integrity of soft tissue and muscles. However, it is challenging to adequately lift proximal femur and broach femoral canal and to avoid femur fracture stem malalignment compared to the posterior approach, leading to the risk of excessive anteversion of the acetabular cup when the surgeon employs DAA in the early stage [22]. The risk of dislocation after DAA has been reported to range from 0.5 to 3% [23–26]. Intraoperative fluoroscopy-guided acetabular component placement was deemed feasible [27, 28], as the use of fluoroscopy guidance offered favorable acetabular component positioning in an anterior approach THA, as described by Gosthe [29] et al. In this study, the number of intraoperative fluoroscopies was reduced in the AIHIP system group compared to the 2D template group, which could not accurately measure the anterior–posterior acetabular diameter [4]. The AIHIP system can adjust the anteversion and abduction angles of the cup based on the 3D model and calculate the acetabular cup coverage simultaneously, which has significant benefits in comparison with the 2D template in accurately predicting the size and anteversion of acetabular prosthesis, ultimately reducing the number of intraoperative fluoroscopic views.

This study demonstrated shorter operative time and less intraoperative bleeding in the AIHIP group compared to the 2D template group. The reduction in operative time may be attributed to decreased intraoperative fluoroscopy and faster prosthesis placement, while the decrease in operative time may contribute to reduce intraoperative bleeding. Intraoperative fluoroscopy can aid in positioning the prosthesis and evaluating its placement, but the C-arm machine takes time to position and fluoroscope, which can result in longer operative times [30]. AIHIP can accurately construct the anatomy of the hip joint and plan the size of prosthesis to assist the surgeon in planning the position and angle of the prosthesis preoperatively, thereby reducing the time for intraoperative prosthesis adjustment and the number of fluoroscopic views and ultimately shortening the operation time. Xia et al. [6] utilized AIHIP to assist in hip replacement in patients with Crowe type IV DDH and demonstrated that AIHIP was effective in assisting the surgeon in filing the acetabulum and selecting the type of prosthesis, which improved the efficiency of the procedure. These findings align with the results of this study. On the other hand, AIHIP improves surgical efficiency and reduces surgical time, which helps to reduce surgical trauma and promote early recovery and functional exercise, which may be the reason for the higher early HSS score of patients in AIHIP group.

Postoperative leg length difference (LLD) is a crucial indicator for evaluating the success of total hip arthroplasty (THA) surgery [31, 32]. Previous literature has reported that patients can perceive a leg length difference when it exceeds 5 mm [33]. LLD is considered the leading cause of postoperative pain, gait disturbance, and aseptic loosening [34–36], and in severe cases, it can even lead to prosthesis revision [37, 38]. It has been reported that there are differences in the morphology of the proximal femur across different regions and age groups, making it challenging for a common femoral prosthesis model to adapt to various hip joints [35]. Boese et al. [39] observed that only 25% of the implants were less than 2 mm away from the medial interface of the femur, and an excessive distance could result in a loosened prosthesis. Some studies have indicated that the morphological variation of the proximal femur [35] and the lack of surgical experience [40] are central factors contributing to postoperative LLD. The literature reported different rates of LLD after DAA, ranging from 8 to 40.4% [34, 41, 42]. The AIHIP system can calculate the bone marrow cavity diameter, leg length difference, and plan the osteotomy height through artificial intelligence (AI) based on high-precision reconstruction of the femoral structure, thereby reducing the postoperative leg length difference. The results of this study showed that the AIHIP group exhibited a reduction in postoperative leg length difference compared to the 2D template group, and the AIHIP system can restore the length balance of both lower extremities to a certain extent.

Femoral offset plays a vital role in the recovery of hip abductor strength after surgery [43–45]. Wu et al. [4] reported that the use of AIHIP positively influenced the postoperative recovery of offset; however, no significant difference was found in this study, which may be explained by different surgical approaches. Further research is urgently needed to confirm the recovery of femoral offset by AIHIP.

This study has some limitations. Firstly, the AIHIP system-assisted planning requires a CT scan, which increases the radiation dose to the patient. Secondly, this study only compared the AIHIP system with 2D templates for preoperative planning, lacking a comparison with 3D planning software. Thirdly, there is a lack of long-term follow-up data on hip joint activity and prosthesis revision.

## Conclusion

The utilization of AIHIP-guided direct anterior approach THA demonstrates higher accuracy and reliability in predicting acetabular cup and femoral stem dimensions, as well as prosthesis implantation location, compared to the traditional 2D planning method. Additionally, it shortens operative time, reduces intraoperative bleeding, and better restores bilateral lower limb length, thus exhibiting greater clinical application value.

## Data Availability

The datasets used during the current study are available from the corresponding author on reasonable request.
